# NKT and NKT-like Cells in Autoimmune Neuroinflammatory Diseases—Multiple Sclerosis, Myasthenia Gravis and Guillain-Barre Syndrome

**DOI:** 10.3390/ijms22179520

**Published:** 2021-09-01

**Authors:** Michał K. Zarobkiewicz, Izabela Morawska, Adam Michalski, Jacek Roliński, Agnieszka Bojarska-Junak

**Affiliations:** Department of Clinical Immunology, Medical University of Lublin, 20-093 Lublin, Poland; 51977@student.umlub.pl (I.M.); 53623@student.umlub.pl (A.M.); jacek.rolinski@umlub.pl (J.R.)

**Keywords:** NKT, iNKT, multiple sclerosis, myasthenia gravis, EAE, MS

## Abstract

NKT cells comprise three subsets—type I (invariant, iNKT), type II, and NKT-like cells, of which iNKT cells are the most studied subset. They are capable of rapid cytokine production after the initial stimulus, thus they may be important for polarisation of Th cells. Due to this, they may be an important cell subset in autoimmune diseases. In the current review, we are summarising results of NKT-oriented studies in major neurological autoimmune diseases—multiple sclerosis, myasthenia gravis, and Guillain-Barre syndrome and their corresponding animal models.

## 1. NKT Cells

**Figure 1:**
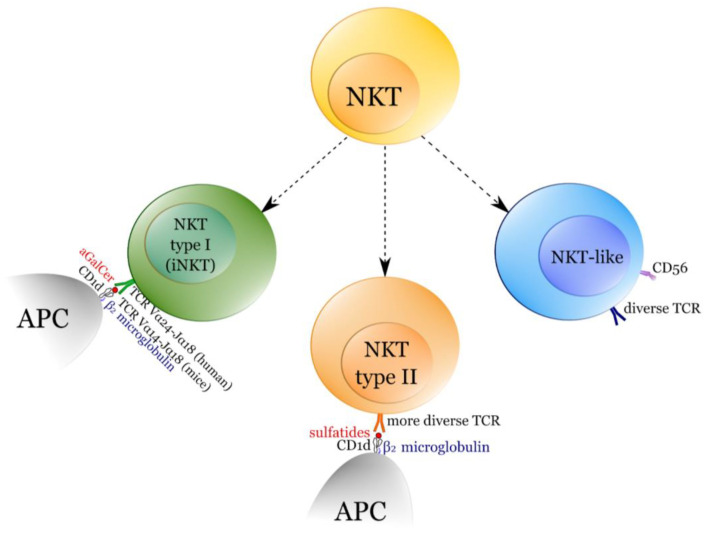
Main populations of NKT cells—type I (invariant NKT, iNKT), type II, and NKT-like. APC—antigen-presenting cell, NKT—natural killer T cell.

NKT cells are classically described as a subset of T cells sharing characteristics of NK cells and typical αβ T cells. NKT cells recognise lipid and glycolipid antigens presented in the context of CD1d molecules, a non-classical MHC molecule [1]. Typically NKT cells are divided into three distinct populations—classical type I NKT (termed also invariant NKT, iNKT), type II (non-classical) NKT, and NKT-like cells [2]. Type I and type II are CD1d-dependent while NKT-like are independent. iNKT cells express a restricted type of TCR (Vα24Jα18 Vβ11 in human, and Vα14Jα18 and Vβ8.2, Vβ7, or Vβ2 in mice), type II NKT cells express a wider array of TCR chains while the NKT-like has even more diverse TCR [1]. iNKT cells recognise α-galactosylceramide (α-GalCer) and some similar lipid-derivatives such as microbial α-glycuronylceramides or human self-antigen isoglobotrihexosylceramide [3]. Moreover, they recognise various microbial antigens derived from microorganisms such as M. tuberculosis, B. burgdorferi, Aspergillus sp., and S. pneumoniae [4]. Type II NKT are activated by the CD1d-sulfatide complex, sulfatide is widely expressed in the kidney, liver, and central nervous system with sulfatide being one of the myelin sheath components [5,6]. Moreover, type II NKT also responds to various antigens of bacterial origins, e.g., M. tuberculosis-derived glycolipids or L. monocytogenes-derived phosphatidylglycerol [6]. Type II NKT cells are also much harder to identify in samples than type I and NKT-like, therefore, they are rarely studied, especially in humans [7]. Major subpopulations of NKT cells are briefly summarized in Figure 1.

iNKT cells are capable of rapid cytokine production early during inflammation, thus providing conventional Th cells with a much-needed cytokine milieu [2,8]. Based on the cytokine profile, iNKT cells can be divided, similarly to conventional Th cells, into iNKT1, iNKT2, iNKT10, iNKT17, iNKTreg, and iNKT_FH_ [9,10,11]. Moreover, those subsets differ in terms of TCR complex expression and signalling strength—iNKT1 having low, iNKT17 intermediate, and iNKT2 high expression/signalling strength [9]. iNKT, especially CD8^+^ and double-negative subsets, are capable of cellular cytotoxicity, utilising two pathways—through cytotoxic granules (perforin, granzymes, and granulysin), and by death receptors (FasL, TRAIL, and TNF) [12]. The cytotoxicity of iNKT cells can be both protective and pathogenic depending on the disease [12]. 

There are some important differences between human and murine NKT cells e.g., up to 50% of human iNKT cells express CD8α while CD8 is nearly non-existent in mouse iNKT cells that are either CD4^+^ or double-negative [2]. On the other hand, iNKT derived from human, mice and non-human primates similarly respond to α-GalCer [3]. Both human and murine iNKT cells are mostly tissue-resident with relatively large populations in lungs, liver, and spleen [13]. Still, there are significant differences in terms of iNKT cells between various strains of mice—C57BL/6 and PL/J have iNKTs comparable in numbers and functions, but SJL/J have very little iNKT cells with severely reduced functionality [14]. Similarly, NOD mice are known to harbour functionally-defective iNKT cells [15]. Finally, iNKT cells usually consist less than one percent in human peripheral blood, while in mice, it is usually 1–2% [16]. 

iNKT cells are probably one of the main responders to HBV infection—they get activated within hours, while conventional Th and Tc cells need 5–6 weeks [13]. To some extent, both type I and type II NKT cells are involved in the pathogenesis of ulcerative colitis and Crohn disease, but their role is not yet fully understood [17]. NKT-like cells are expanded in solid tumours, which is probably due to the disease as, after complete remission, their percentage returns to reference values [18]. NKT-like cells are also probably involved in the pathogenesis of chronic obstructive pulmonary disease (COPD) [19]. The role of type II NKT cells in autoimmunity is still to be fully understood—in some cases, they may be protective while in others being highly detrimental [6].

## 2. Multiple Sclerosis

Multiple sclerosis is a chronic demyelinating disease of the central nervous system (CNS) with complex and not yet fully understood etiologically. Although no conclusive epidemiological data are available, it is estimated that the prevalence of the disease in Europe is around 83 per 100,000 inhabitants [20]. Nevertheless, according to a recent study by Walton et al., prevalence of MS in Germany may be higher than 200 per 100,000 inhabitants with a European average of 143 [21]. The pathophysiology of multiple sclerosis is very complex and involves several key elements such as damage to the blood-brain barrier, multifocal inflammatory process within the CNS that leads to demyelination, secondary astroglial hyperplasia, and axonal damage within myelinated neuronal pathways [22]. Autoreactive T cells are suspected to play a significant role in inducing tissue inflammation in MS [23]. 

### 2.1. iNKT in MS

The percentage of iNKT cells was significantly increased in peripheral blood (PB) of untreated RRMS patients compared to healthy volunteers [24,25] but not in SPMS or PPMS patients [25]. On the other hand, a significant decrease, more pronounced in remission than relapse, was noted in another study, this can be attributed mostly to a decrease in double-negative (CD^4−^/CD^8−^) and CD^8−^ subsets, the CD4^+^ subpopulation seems to be unaffected [26]. An overall decrease in circulating iNKT cells was observed in MS, but also in a number of different autoimmune diseases that primarily focus on tissue destruction [27]. Similarly, a significant decrease in iNKT cells among total PBMCs was observed on mRNA level (Vα24 transcript) in relapse compared to healthy controls (HC) [28,29]. That does not necessarily mean, however, reduction in iNKT percentage—different expression levels of TCR is being constantly observed in flow cytometry, thus it is possible that MS patients have decreased expression of TCR on iNKT cells. Moreover, a significant decrease in the diversity of Vα24 transcript was observed in relapse MS patients compared to healthy controls [28,29]. An increase was, however, observed after treatment with recombinant IFN-β in a group of patients well responding to this therapy [24]. The former stays in contrast to our results—we observed no significant difference in peripheral blood iNKT percentage between patients in remission or relapse of RRMS and healthy volunteers [30]. Nevertheless, we have observed functional differences—overexpression of RORγT and IL-23R in iNKT both during relapse and remission of RRMS, moreover, we noted a significant rise in iNKT17 during remission. Preliminary data of our current ongoing immunophenotyping study confirm overexpression of RORγT and suggest a decrease in T-bet and increase in GATA3 expression (possible skew towards iNKT2 subset). Similarly, CD4^+^ iNKT cells were previously observed to be skewed towards Th2-type response, in terms of cytokine production, in MS compared to HC, in the same time the CD^4−^ iNKT subsets revealed neither Th1- nor Th2- predominance [26]. Finally, double negative iNKT cells seem to produce both IL-4 and IFN-γ; though, a significant decrease in IL-4 producing double negative iNKT cells was noted in RR-MS patients compared to both healthy volunteers and chronic progressive MS [31].

On the other hand, iNKT cells from MS patients seemed to not respond to stimulation with α-GalCer, either by proliferation or by the production of IFN-γ [25], a similarly weaker proliferation was observed by others [26]. This was, however, contradicted by a study of Gigli et al., who observed a significant increase in IFN-γ, IL-4 and IL-5 production by α-GalCer-stimulated iNKT after IFN-β treatment [24]. The effect of IFN-β was mediated by dendritic cells—direct addition of IFN-β to cell culture completely blocked cytokine secretion in iNKT cells [24]. Blocking the activity of phosphodiesterase with non-selective inhibitor ibudilast promoted a significant increase in iNKT percentage among T cells in RR-MS patients as well as promoting a general Th2-shift in Th cells in peripheral blood [32].

The extent to which iNKT cells are involved in the pathogenesis of MS may nevertheless be limited as iNKT cells seem to be rarely present in higher numbers in MS plaques and CSF [29]. Due to the complicated nature of MS pathogenesis and relatively low abundance of iNKT in general, no firm conclusions can be drawn from human studies. 

### 2.2. NKT-like

There was no difference in the percentage of NKT-like (CD3^+^/CD56+) lymphocytes between MS (both RRMS and PPMS) and healthy subjects [33,34] as well as between various forms of MS (RRMS, PPMS, and SPMS) [25]. On the other hand, according to Jons et al., the percentage of NKT-like cells in both peripheral blood and bone marrow is significantly lower in MS patients compared to healthy subjects [35]. 

Similarly, no significant difference was observed in the expression of various adhesion molecules (LFA-1, ICAM-1, ICAM-3, and VLA-4) on the surface of NKT-like cells between healthy volunteers and treatment-naive RRMS patients [36]. During glatiramer acetate treatment, the expression of ICAM-1 and ICAM-3 decreased—the former after 1.5 month, the latter required a year-long treatment [36]. The percentage of NKT-like (CD56dim/CD3^+^) cells was decreased in CSF of RRMS patients compared to non-inflammatory controls (non-inflammatory neurological disease) [37,38], but it did not differ from inflammatory control (inflammatory neurological disease) [38]. No difference in NKT-like cells was noted in peripheral blood [38]. Nevertheless, NKT-like in CSF (absolute numbers) are also significantly increased in active MS compared to stable MS [38]. A significantly higher activation rate (CD69^+^) was observed among CD8^+^ NKT-like cells in MS pregnancy compared to healthy control pregnancies and non-pregnant MS women [39]. Similarly, among men, there was a significantly higher activation rate in MS than HC [39]. Together, it all suggests just a minor role of NKT-like cells in MS. 

### 2.3. Effect of Treatment on NKT-like Cells

Natalizumab and fingolimod are two highly effective drugs for RRMS. Natalizumab is a monoclonal antibody anti-α4 integrin; it blocks interactions of α4 integrins with their endothelial receptor, thus lowering the migration of lymphocytes to CNS [40]. Fingolimod is a small molecule blocker of sphingosine-1-phosphate receptors, and as an effect, it blocks the migration of lymphocytes from lymph nodes into periphery [41]. Natalizumab binds, to a significantly higher degree, to NKT-like cells and NK cells than conventional T cells and B cells, which seems to be directly related to higher expression of α4 integrin [42]. Nevertheless, long-term natalizumab treatment seems to not affect PB type NKT-like counts [42]. Fingolimod seems not to change NKT-like absolute numbers in PB, but an increase in NKT-like percentage is observed—that, however, is most probably a result of a decrease in absolute numbers of T and B lymphocytes [33]. Finally, in a subgroup of patients receiving dimethyl fumarate who developed lymphopenia, a significant (approx. a 2-fold) decrease in NKT-like percentage was observed [43]. Similarly, after oral cladribine treatment NKT-like cells were significantly decreased in peripheral blood (approx ⅕ of baseline value) [44]. The data on the influence of various treatment regimens on NKT-like cells is severely limited and although some regimens clearly affect NKT-like subset, it is impossible to say whether that is a side effect or it is important for clinical efficacy. 

## 3. Experimental Autoimmune Encephalomyelitis (EAE)

Experimental autoimmune encephalomyelitis is a widely used murine model of multiple sclerosis [45]. EAE is most commonly induced by immunisation with either myelin basic protein (MBP) or part of myelin oligodendrocyte protein (MOG35-55), in both cases along with adjuvant [45]. The course of EAE depends on both the immunisation protocol and species and strain of animal [45]. Although studies on EAE pathogenesis are important for our understanding of MS, every such study should be analysed with caution due to important differences between them, e.g., MS is a chronic inflammatory condition, while EAE tends to be self-limiting after single relapse [46].

Nevertheless, use of EAE enabled a more in-depth understanding of iNKT involvement in multiple sclerosis pathogenesis. First of all, it allowed for studying the disease in the total absence of iNKT cells. In fact, a more severe course of EAE was observed in iNKT-deficient mice; adoptive transfer (before immunisation) of iNKT led to decreased symptoms [47]. Similarly α-GalCer stimulation attenuated EAE symptoms [47]. α-GalCer administration protects against EAE, but only in mice with significant iNKT population—there was scarcely any effect in SJL/J mice, moreover, an increase in mortality was observed in those mice [14]. Nevertheless, multiple different methodological approaches and mice strains used significantly complicate the picture. On the one hand, no difference in EAE symptoms and severity was observed between wild-type and β2-microglobulin-knockout mice (lacking iNKT cells) [48]. On the other hand, iNKT-knockout mice (Jα1^8−^knockouts) develop significantly more severe EAE course [49]. This points, clearly, to the importance of methodology applied. Moreover, functional state and expression of surface receptors seem to play a role as well. GPR65 expression in iNKT cells seems important as mice that lack GPR65 in iNKT develop exacerbated disease, GPR65-deficiency of conventional T cells does not make any significant difference [50].

As normal NOD mice have significantly impaired iNKT functions, complete iNKT knockout in NOD mice does not affect severity of EAE [15]. Transgenically induced increases in iNKT numbers leads, however, to milder and delayed EAE in NOD mice and corresponding lower CNS infiltration without signs of perivascular demyelination [15]. Nevertheless, the percentage and function of isolated lymph-node MOG-specific T cells seems to be unchanged. Possibly because lymph nodes contain relatively low numbers of iNKT cells—in spleen (relatively high iNKT number) a significant downregulation of IFN-γ production by MOG-specific T cells was noted [15]. Those effects seem to be IL-4-independent—IL-4 deficient iNKT-enriched mice have similarly diminished disease severity [15].

Similarly, expanded and adoptively transferred iNKT cells protect against full-spectrum EAE, even with extrathymic CD1d knockout [51]. Moreover, encephalitogenic Th cells are also suppressed by iNKTs in a CD1d-independent manner [51].

iNKT cells can infiltrate CNS, and the highest number was found on day 21 after immunisation; α-GalCer further increases that infiltration [47]. CNS-infiltrating iNKT cells are predominantly double negative (CD4^−^/CD8^−^) and produce mostly IFN-γ, IL-17, and granzyme B [51].

**Figure 2:**
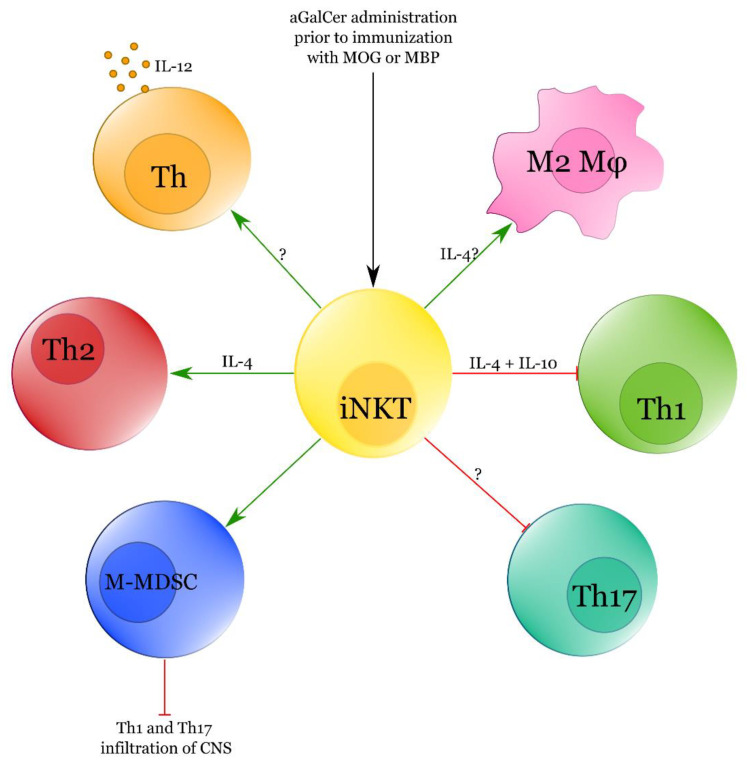
Major modes of action of iNKT cells in EAE. Red lines are for inhibitory while green ones for promoting interactions. *M-MDSC—monocytic myeloid-derived suppressor cells. Th—T helper cells, M2 Mφ—M2 macrophages*.

Major studies on iNKT cells in EAE are briefly summarised in Table 1, while major interactions of iNKT cells with other cellular subsets in EAE are presented in Figure 2. 

### 3.1. iNKT-Mediated Changes in Cytokine Milieu

It seems that timing of α-GalCer injection is a crucial thing for the outcome. Co-immunisation with MBP and α-GalCer exacerbates EAE by promoting IFN-γ production, while pre-immunisation decreases symptoms by upregulating IL-4 secretion [52]. To complicate it—administration of α-GalCer shortly before or after (but not together) MOG35-55 immunisation does not induce any significant changes in EAE course or severity [60]. The question emerges—what is the mode of action of α-GalCer. Moreover, α-GalCer treatment in IL-4 knockout mice increased severity, while in IFN-γ-knockout ones decreased the severity [60]. Similarly, use of synthetic analogue of α-GalCer, that induces IL-4 production without promoting IFN-γ in iNKT cells, suppresses development of EAE [53], thus suggesting the importance of IL-4 produced by iNKT cells in disease suppression. Moreover, α-GalCer mediated activation of iNKT cells seem to promote a shift towards Th2-type response to MOG35-55 [60]. iNKT cells activated by α-GalCer promote M-MDSC accumulation during EAE, especially notable in the 2nd week after induction [54]. It is primarily mediated by cytokines, not direct contact—while GM-CSF seems to be important for expansion while IL-4 and IFN-γ are important for promoting immunosuppressive potential in M-MDSC [54]. Moreover, those M-MDSC infiltrated the spinal cord; consecutively, a significantly lower number of Th cells, including Th1 and Th17, in the spinal cord was noted [54]. On the other hand, according to Furlan et al., CFA-α-GalCer diminishes severity and delays onset of EAE by activation on iNKT and further upregulation of IFN-γ with very little IL-4 production by iNKT [55]. Moreover, neutralisation of IFN-γ completely reverses those effects [55]. Finally, CFA-α-GalCer stimulated iNKT do not affect MOG35-55-specific Th cells, but promote IL-10 production by those cells [55]. Similarly, in C57BL/6 mice, α-GalCer lowers IFN-γ production and increases IL-10 in Th cells from lymph nodes; in PL/J mice, it also increases IL-4 production [14]. No protective effect of α-GalCer was noted in either IL-4 or IL-10 deficient mice [14]. iNKT cells inhibit production of IL-17 and IFN-γ by Th cells [47]. IL-4 and IL-10 seem crucial for Th1 inhibition by iNKT cells, but they are irrelevant for Th17 inhibition; moreover, IFN-γ producing iNKT seems to be irrelevant for iNKT-mediated EAE suppression [47]. Injection of MOG-pulsed TNF-pretreated dendritic cells protects against EAE by activating iNKT cells and promoting Th2-like response thereof [57]. Those iNKT not only produce IL-4 and IL-13, but they also promote Th2-type response among conventional Th cells, as well as development of MOG-specific IL-10-producing Th cells [57]. It seems that this process requires direct interaction between DCs and conventional Th cells as well as Th2-related cytokines produced initially by activated iNKT cells [57]. Altogether, it suggests that a proper activation of iNKT cells done at the right time promotes IL-4 production that further primes conventional Th cells to Th2 and Treg phenotypes that, at least partially, protect against EAE.

### 3.2. Gut Microbiota and iNKT in EAE

The importance of gut microbiota in MS and EAE pathogenesis is still not fully understood. Still, gut microbiota seems to play some role in it [61]. Indeed, composition of gut microbiota may partially regulate the susceptibility of mouse to EAE—severe alteration of gut microbiota by combination of kanamycin, colistin, and vancomycin was found to effectively prevent the establishment of EAE by reducing Th17 cells in mesenteric lymph nodes [62]. This effect was not observed in iNKT-deficient mice, thus implying the importance of iNKT cells in this process [62]. The exact role of iNKT in this phenomenon remains to be studied.

### 3.3. Vitamin D and iNKT Cells in EAE

Growing body of evidence suggests involvement of vitamin D in MS pathogenesis [63]. Indeed, vitamin D3 protected wild-type mice (but not CD1d-knockout mice) against EAE by promoting IL-10 and decreasing IL-17 and IFN-γ production in lymph nodes [58]. Moreover, administration of α-GalCer rendered similar protection against EAE [58]. Vitamin D3 and α-GalCer action seems to be IL-4-dependent [58]. Moreover, iNKT cells are involved as vitamin D3 was significantly less efficient in protecting against EAE in iNKT-deficient mice [58]. In contrast to α-GalCer, Vitamin D3 significantly lowers iNKT-infiltration of CNS [58]. Co-stimulation with α-GalCer and vitamin D3 increased IL-4, IL-5 and IL-10 secretion by iNKT compared to sole α-GalCer stimulation [58]. Moreover, vitamin D3 significantly lowered IL-17 and IFN-γ production in mice MOG-specific splenocytes [58]. iNKT cells, especially after activation with α-GalCer seem to promote transition of macrophages into M2 by secreting IL-4, thus lowering the severity of EAE [49]. Antibody-mediated IL-4 neutralisation seems to completely cancel the positive effect of α-GalCer stimulation on macrophage polarisation [49]. Results from in vitro studies on Crohn disease, and healthy volunteer samples, imply that vitamin D promotes M2 over M1 macrophages and lowers their production of pro-inflammatory cytokines [64,65]. Thus, it is possible that this positive effect of α-GalCer and vitamin D3 may both be a result of change within macrophages, at least to some extent.

### 3.4. NKT Type II in EAE

Sulfatide reactive NKT cells were observed in CNS during EAE, but not in control mice [5]. Moreover, they are 3–4 times more numerous than iNKT cells therein during EAE. Those type II NKT cells secrete, predominantly, IFN-γ [5]. Co-administration of sulfatide with MOG35-55 exerts a strong protective effect, which is CD1d dependent [5]. Similar protection was observed when sulfatide was given one week prior or post MOG35-55-immunisation [5]. Sulfatide treatment lowers the number of MOG35-55 reactive T cells, both Th1 and Th2 [5]. On the other hand, epicutaneous immunisation with myelin basic protein (MBP) leads to significant suppression of EAE and this effect is much more pronounced in CD1d-deficient animals [66]. Still, a more severe EAE, with tendency towards chronic course and higher demyelination, was observed in CD1-deficient mice [59]. A significantly lower TGF-β-production by T cells at the end of acute phase, and higher production of IFN-γ and IL-4 by autoreactive T cells was noted in those CD1d-deficient animals [59]. 

Administration of bovine-brain-derived sulfatides without adjuvant after establishment of EAE leads to significantly reduced symptoms, adding adjuvant leads to exaggerated symptoms [56]. That positive effect was even more pronounced when, instead of a mixture, only one single (most potent) sulfatide—*cis*-tetracosenoyl sulfatide—was given [56]. It suggests that activation of type II NKT cells during an already established course significantly lowers disease burden [56]. Such treatment also leads to 10-fold (lymph nodes) or 3-fold (CNS) reduction in encephalitogenic Th cells in a PLP139-151 induced EAE [56]. Moreover, among those PLP139-151-reactive cells, a notable reduction in IL-17 and IFN-γ production was noted in groups treated with sulfatides, but when sulfatide was administered with adjuvant, a notable rise in IL-17 production was observed. This explains why such a mixture does not decrease EAE symptoms [56]. Co-administration of sulfatide with PLP139-151 and adjuvant may even lead to a more severe EAE course [67]. Sulfatide-activation of type II NKT also induced anergy of iNKT cells. This seems crucial for a sulfatide-mediated decrease in EAE symptoms, as iNKT-deficient mice do not show any improvement after sulfatide-treatment [56]. This may further suggest that those anergic iNKT cells play a regulatory-like role [56]. Moreover, a 3-fold decrease in CNS-infiltrating iNKT cells was noted [56]. A decrease in expression of CD1d, MHC class II and activation markers (CD80/86) on microglial and CNS-infiltrating macrophages was also noted after sulfatide treatment [56]. Treatment with α-GalCer at the time of the onset of EAE symptoms does not alter disease course in SJL/J mice [56]. Results and methodology of the major significant studies on type II NKT in MS/EAE are summarised in Table 2.

## 4. Myasthenia Gravis (MG)

Myasthenia gravis (MG) is a chronic, immune-related neuromuscular disease with presence of autoantibodies against nicotinic acetylocholine receptors (AChR), muscle-specific kinase (MuSK) autoantibodies to low-density lipoprotein receptor-related protein 4 (Lpr4) or remains seronegative [69,70]. Although most cases are sporadic, there is an observation of familial susceptibility to the MG [71,72,73]. Disease is diagnosed in 0.25–2 per 1 million people, mostly in women under the age of 40 and rarely in children [74,75,76]. Due to receptor blocking or destruction signal transduction through neuromuscular junction can be severely impaired. As a result, patients might present skeletal muscle weakness, from isolated ptosis to lethal symptoms due to the loss of respiratory muscle function [77,78]. MG frequently coexists with thymic hyperplasia or thymoma [79,80,81]. The pathogenesis of MG is not yet fully understood and it is suspected that genetic, environmental, infectious, and immunological factors are involved [82,83,84,85,86,87,88,89]. Based on the recent studies, as well as the experimental autoimmune MG (EAMG), it is postulated that thymus tissue is able to create an autoimmune-microenvironment and plays a crucial role in the outcome of the disease [90].

### iNKT in MG

Proper functioning of thymus-derived iNKT plays a main role in peripheral immunological tolerance, which is necessary to strive for the development of autoimmunity [91]. Decrease in iNKT numbers have been found in many autoimmune diseases as well as in mice models of autoimmunity [92,93,94,95]. Although in most autoimmune diseases, as well as in their animal models, the number of invariant NKT levels is usually decreased, in myasthenia gravis (MG) an increased amount of iNKT was observed [27]. Increased iNKT numbers in MG patients significantly dropped after treatment [96]. Moreover, MG patients with thymic hyperplasia (but not with thymoma) have even higher percentages of NKT cells in the peripheral blood [97]. Jα281^−/−^ and CD1d^−/−^ (without both I and II type NKT presence) mice have normal susceptibility to EAMG in comparison to the healthy ones [98,99,100]. It is hypothesised that iNKT cells are functionally involved in establishing the immune tolerance and defect of NKT-cells may lead to outcome of the disease, but they are not pathogenic in MG by themselves. In the animal model, stimulation of iNKT results in IL-2-dependent increase in expansion of CD4^+^CD25+Tregs and prevention of EAMG outcome. The IL-2 mRNA and IL-2 expression in activated NKT cells, in that study, was significantly higher than in the control group [99,101]. α-GalCer administration in mice results in up-regulation of expression of antiapoptotic bcl-2 and FoxP3 proteins in CD4^+^CD25+ cells and, as a result, enhancing their functioning. Those CD4^+^CD25+ cells had better proliferative properties and were more potent to inhibit autoreactive T-cells [99]. What is interesting is, in MG, there is a postulated defect in the functioning of CD4^+^CD25+Tregs [95].

NKT cells are able to quickly release great amounts of cytokines, both pro- and anti-inflammatory, such as IL-2, IL-10, IL-17, IL-4, and IFN-γ upon the stimulation, but without the need for prior activation [102,103]. The influence of various cytokines on the development of EAMG is not entirely clear, however, it has been suggested that IFN-γ (mainly related to the Th1 response) influences the development of EAMG and, surprisingly, IL-4 (mainly related to the Th2 response) does not play as significant role in preventing EAMG as it was speculated [104,105]. Interestingly, in the mice model of MG, treatment with α-GalCer lowered the production of IFN-γ but did not interfere with IL-4 level, suggesting the important regulatory role of iNKT [99]. IL-4 deficient mice response to the α-GalCer treatment was statistically the same as wild-ones. Most importantly, CD1d^−/−^ mice (without the NKT cells) did not respond to glycolipid treatment and that strongly proves the importance of NKT cells in preventing MG outcome. Because of the fact that α-GalCer is able to activate both murine and human NKT cells, the experimental findings on this therapy in autoimmune diseases may be translatable from experimental to human models [103].

## 5. Guillain-Barré Syndrome

Guillain-Barré syndrome (GBS) is the most common acute disorder of the peripheral nervous system (PNS), characterised by accumulation of autoreactive T cells and macrophages in the PNS as well as demyelination [106]. GBS can be divided into demyelinating type, with acute inflammatory demyelinating polyneuropathy being the most common manifestation of GBS, and axonal type, mediated by anti-GM1, anti-GD1a, anti-GT1a, and anti-GQ1b IgG autoantibodies [106,107]. Most commonly, the onset of this disorder is preceded by the infection, especially in a case of Campylobacter jejuni, or other immune system stimuli, resulting in an autoreactive response that targets both peripheral nerves and their spinal roots [108].

In pathogenesis of GBS, iNKT cells were previously suspected to act as T helper cells, promoting both maturation and IgG-switching of B cells producing anti-ganglioside antibodies [109]. However, it has been proven that, in the course of experimental autoimmune neuritis (EAN), an animal model of GBS, after exposing iNKT cells to lipo-oligosaccharides (LOS) no significant change in production of cytokines was discovered, making it improbable that IgG anti-LOS response is mediated by iNKT cells [110]. No significant difference between GBS and non-inflammatory control was observed for NKT-like percentage in CSF [37].

As already discussed, sulfatides can significantly modulate the course of EAE and, consistently with that, administration of sulfatide caused both amelioration of EAN symptoms and suppression of lymph node NKT cells, which include both type I and type II NKT cells. Simultaneously, treatment with sulfatides resulted in a significant decrease in NKT type I and II cells in lymph nodes [111]. Moreover, administration of sulfatides resulted in inhibition of Th and antigen presenting cells, as well as T cell proliferation and IL-17 production [111]. Altogether, it seems that type II NKT cells may have important protective capacity in EAN and thus, possibly, also in GBS.

## 6. Conclusions

Both iNKT and NKT type II cells are complex and heterogeneous cell subsets. Although their biology is not yet fully understood in general, they seem to be important players in the pathophysiology of autoimmune neuro-inflammatory diseases, e.g., multiple sclerosis. 

Moreover, some pre-clinical studies on the murine models suggest that iNKT may be specifically targeted to treat autoimmunity, including multiple sclerosis. The wider perspectives for iNKT-mediated autoimmunity treatment were briefly summarised by Van Kaer and Wu [112]. Most importantly, α-GalCer, which was so widely used in animal model studies, seems to not be a potential drug for iNKT modulation. Moreover, iNKT can possibly be expanded in vitro and then subsequently transferred to the patient [113]. To summarise, iNKT cells provide some interesting opportunities for the development of novel treatment regimens for autoimmune diseases, provided that, with future studies, our understanding of iNKT biology will sufficiently improve.

## Figures and Tables

**Table 1:** Involvement of iNKT in EAE—major studies.

No	Study Design	Major Results	Citation
1	Female B10.PL and C57BL/6 mice + CD1d or IFN-γ knockouts; immunisation with either MBP or MOG35-55; co-immunisation with α-GalCer at different time points	Co-immunisation with MBP and α-GalCer further promotes Th1 phenotype in T cells and significantly exacerbates EAE. Pre-immunisation alleviates EAE symptoms by promoting IL-4 over IFN-γ production.	Jahng et al., 2001 [52]
2	C57BL/6 mice. Synthetic analogue of α-GalCer administered along with MOG35-55	Suppression of EAE by promotion of IL-4 production in iNKT cells	Miyamoto et al., 2001 [53]
3	C57BL/6 mice. MOG35-55 induced EAE and adoptive transfer of MOG-speciifc T cells with co-administration of α-GalCer	α-GalCer activated iNKT cells promote M-MDSC expansion, lowering symptoms of EAE	Parekh et al., 2013 [54]
4	C57BL/6 mice and knockout, CFA+ α-GalCer stimulation. MOG35-55 induced EAE	iNKTs are not necessary for establishment of EAE. Activation of iNKT diminishes severity and delays onset of EAE probably through IFN-γ increase	Furlan et al., 2003 [55]
5	NOD mice, transgenic enrichment of iNKT cells and extrathymic CD1d knockout; EAE induced with MOG35-55	iNKTs diminish the severity of EAE. DN, cytotoxic iNKT cells infiltrate CNS. CD1d seems not necessary for iNKT mediated protection	Mars et al., 2008 [51]
6	C57BL/6 and knockouts (iNKT, IL-4, IL-10, IFN-γ), MOG35-55 induced EAE, co-administration of α-GalCer (day before and day after); adoptive transfer of iNKT	iNKTs inhibit Th1 and Th17 response; the former is mediated by IL-4 and IL-10.	Oh and Chung, 2011 [47]
7	SJL/J and C57BL/6 female mice, transgenic iNKT-deficient C57BL/6 mice; EAE induction with either PLP139-151 or MOG35-55; adoptive transfer (2 days prior to immunisation) of liver dendritic cells from sulfatide-pretreated mice	Activation of type II NKT by sulfatides after EAE is established leads to amelioration of symptoms, probably due to induction of anergy in iNKT cells, rendering more regulatory phenotype of iNKT and reducing number of CNS-infiltrating iNKT cells. Moreover, it also leads to decrease in encephalitogenic total Th as well as Th1 and Th17 cells.	Maricic et al., 2014 [56]
8	C57BL/6 mice, various knockout mice and iNKT transgenic mice. EAE was induced with MOG35-55. Adoptive transfer of dendritic cells at various time points before immunisation, adoptive transfer of iNKT cells one day prior to first dendritic cell injection.	Injection of MOG-pulsed TNF-pretreated dendritic cells protects against EAE by activating iNKT cells and promoting Th2-like response thereof.	Wiethe et al., 2007 [57]
9	Male Va14-Ja281 and Va8 and transgenic NOD mice; various knockout mice; EAE induction with MOG35-55	Increased iNKT number significantly decreases EAE severity and delays onset in transgenic NOD mice.	Mars et al., 2002 [15]
10	C57BL/6 mice, both male and female as well as knockout mice (CD1d-, Jα18- or IL-4-deficient); MOG35-55-induced EAE; supplementation with active vitamin D3 (1,25-hydroxy-D3). Co-administration of α-GalCer on day of immunisation.	iNKT cells are important mediators of vitamin D3-mediated EAE protection. This effect is at least partially dependent on IL-4.	Waddell et al., 2015 [58]
11	C57BL/6 mice, CD1-knockouts (lacking both iNKT and type II NKT cells), MOG35-55-induced EAE, adoptive transfer	CD1-knockout mice had significantly more severe EAE with a tendency towards more chronic course and higher demyelination. Significantly lower TGF-β production in CD1-deficient mice after acute phase was over.	Teige et al., 2004 [59]
12	C57BL/6 mice, Jα18-knockout, MOG35-55-induced EAE	iNKT-knockout mice develop more severe EAE course. IL-4-produced by iNKT cells seem to be crucial for induction of M2-polarisation of macrophages, thus decreasing EAE severity	Denney et al., 2012 [49]

**Table 2:** Involvement of type II NKT in EAE—major studies.

No	Study Design	Major Results	Citation
1	SJL/J and C57BL/6 female mice, transgenic iNKT-deficient C57BL/6 mice; EAE induction with either PLP139-151 or MOG35-55; adoptive transfer (2 days prior to immunisation) of liver dendritic cells from sulfatide-pretreated mice	Activation of type II NKT by sulfatides after EAE is established leads to amelioration of symptoms, probably due to induction of anergy in iNKT cells, rendering more regulatory phenotype of iNKT and reducing number of CNS-infiltrating iNKT cells. Moreover, it also leads to decrease in encephalitogenic total Th as well as Th1 and Th17 cells.	Maricic et al., 2014 [56]
2	C57BL/6 mice, various knockouts; EAE induced with MOG35-55. Administration of sulfatides either at the same time with MOG35-55 or one week before or after.	Type II NKT cells are present in CNS during EAE and are more prevalent than iNKT. Administration of sulfatide 7 days prior, along with or 7 days after MOG35-55 significantly lowers disease burden	Jahng et al., 2004 [5]
3	C57BL/6 mice and knockout mice (PD-L1-, CD1d or Jα281-deficient); MOG35-55 induced EAE. Adoptive transfer of tolerogenic TNF-pretreated DC either expressing PD-L1 or PD-L1-deficient	Tolerogenic DC, especially those PD-L1 deficient promote production of Th2-type cytokines by type II NKT cells thus decreasing severity of EAE	Brandl et al., 2010 [68]

## Data Availability

Not applicable.
